# Dissemination of carbapenemase-producing *Enterobacteriaceae* harbouring *bla*_NDM_ or *bla*_IMI_ in local market foods of Yangon, Myanmar

**DOI:** 10.1038/s41598-019-51002-5

**Published:** 2019-10-08

**Authors:** Yo Sugawara, Hideharu Hagiya, Yukihiro Akeda, Mya Mya Aye, Hpoo Pwint Myo Win, Noriko Sakamoto, Rathina Kumar Shanmugakani, Dan Takeuchi, Isao Nishi, Akiko Ueda, Moh Moh Htun, Kazunori Tomono, Shigeyuki Hamada

**Affiliations:** 1 Japan–Thailand Research Collaboration Center on Emerging and Re-emerging Infections, Research Institute for Microbial Diseases, Osaka University, Suita, Japan; 2 Department of Infection Control and Prevention, Osaka University Hospital, Suita, Japan; 3 Bacteriology Research Division, Department of Medical Research, Yangon, Myanmar; 4 Laboratory for Clinical Investigation, Osaka University Hospital, Suita, Japan

**Keywords:** Antimicrobial resistance, Bacterial infection

## Abstract

The spread of carbapenemase-producing *Enterobacteriaceae* (CPE) poses a serious threat to clinical practice and public health. These bacteria are present both in clinical settings and non-clinical environments. The presence of CPE in food stuffs has been reported, but sporadically so. Here, we screened for CPE in meat, seafood, and vegetable samples from local markets of Yangon, Myanmar. We obtained 27 CPE isolates from 93 food samples and identified 13 as *Escherichia coli*, six as *Klebsiella pneumoniae*, seven as *Enterobacter cloacae* complex, and one as *Serratia marcescens*. All except the *E*. *cloacae* complex harboured the carbapenemase genes *bla*_NDM-1_ or *bla*_NDM-5_, while all *Enterobacter* isolates carried the carbapenemase gene *bla*_IMI-1_. The *bla*_IMI-1_ gene was located in putative mobile elements EcloIMEX-2, -3, or -8. Using multi-locus sequence typing, *E*. *coli*, *K*. *pneumoniae*, and *E*. *cloacae* complex isolates were classified into 10, six, and five different sequence types, respectively. Our results demonstrate that diverse organisms with various carbapenemase genes are widespread in the market foods in Yangon, highlighting the need for promoting proper food hygiene and effective measures to prevent further dissemination.

## Introduction

Carbapenem-resistant *Enterobacteriaceae* have become a global public-health concern because these bacteria cause high mortality and are multidrug-resistant^1^. This resistance is mainly due to the production of carbapenemases, which hydrolyse carbapenem antibiotics. Carbapenemase genes such as *bla*_NDM_ are often in transmissible plasmids and thus spread readily among *Enterobacteriaceae* species^2^. Currently, carbapenemase-producing *Enterobacteriaceae* (CPE) are found in both clinical and non-clinical settings, including sewage systems^3,4^. Alarmingly, CPE have been isolated from imported food stuffs (vegetables and seafood) originating in southeast Asian countries^5,6^. Despite the potential danger, thorough food surveillance is not performed on retail foods in CPE-endemic regions, with a few exceptions in China^7,8^.

Previously, we identified genetically diverse *Enterobacteriaceae* isolates with *bla*_NDM_-harbouring plasmids in a hospital of Yangon, Myanmar’s largest city^9,10^. We then found closely related strains in sewage water outside the hospital^10^, implying that CPE in clinical settings could reach the external environment. These findings prompted us to investigate the extent of CPE dissemination in local communities. Here, we focused on the spread of CPE in local markets.

## Results

### Diverse *E*. *coli* and *K*. *pneumoniae* isolates harbouring *bla*_NDM_ from market foods

**Figure 1:**
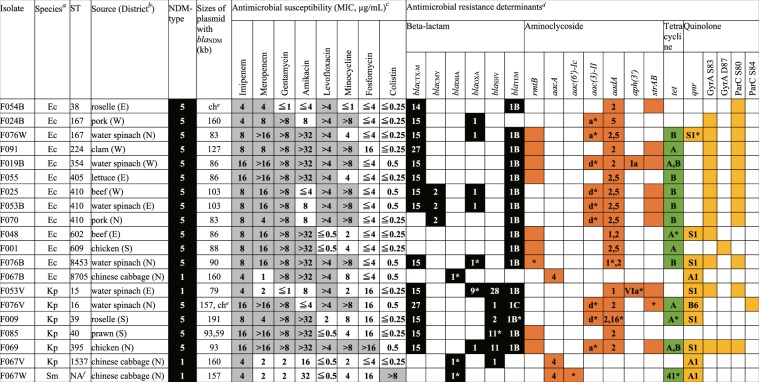
Phenotypic and genetic characteristics of CPE isolates harbouring *bla*_NDM_. ^*a*^Ec, *Escherichia coli*; Kp, *Klebsiella pneumoniae*; Sm, *Serratia marcescens*. ^*b*^Food samples were obtained from southern (S), western (W), eastern (E), and northern (N) districts of Yangon. ^*c*^Grey cells indicate isolates resistant to the indicated antimicrobials. MIC, minimum inhibitory concentration. ^*d*^Numbers and alphabets in columns denote variant types. For GyrA and ParC, presence of amino acid substitution in the indicated residues are marked in dark yellow. Genes with nucleotide substitution(s) compared with sequences in the ResFinder database are marked with asterisks. ^*e*^Southern blots identified *bla*_NDM-5_ gene on chromosomes. ^*f*^NA, not applicable.

Of the 93 food samples from eight markets, we identified 20 CPE-positive samples (21.5%). Within each food category (meat, seafood, and vegetables), the percentage of CPE-positive samples was 25.0% (8/32), 10.3% (3/29), and 28.1% (9/32), respectively (Table 1). We obtained 27 CPE isolates from the 20 samples, with 16 (59.3%) derived from vegetables. Water spinach exhibited the highest CPE-positive rate (4/8, 50%) and yielded eight CPE isolates. Of the 27 CPE isolates, 20 carried *bla*_NDM_, including 13 *Escherichia coli*, six *Klebsiella pneumoniae*, and one *Serratia marcescens*. All but one *E*. *coli*, along with four of six *K*. *pneumoniae*, possessed *bla*_NDM-5_; the remaining carried *bla*_NDM-1_ (Fig. 1). Every *bla*_NDM_-carrying isolate was resistant to imipenem. Those harbouring *bla*_NDM-5_ were also resistant to meropenem, but *bla*_NDM-1_ did not confer resistance to this antibiotic. All isolates were resistant to extended-spectrum cephalosporin and oxacephem antibiotics, such as ceftazidime and moxalactam, and the minimum inhibitory concentration for these antibiotics was >64 µg/mL and ≥128 µg/mL, respectively. Additionally, 80% (16/20), 55% (11/20), and 70% (14/20) of isolates were resistant to gentamycin, amikacin, and levofloxacin, respectively.

**Table 1:** Isolation of CPE from food samples in the markets of Yangon, Myanmar. ECC, *E*. *cloacae* complex; Sm, *S*. *marcescens*.

Source	No. of samples	No. of CPE-positive samples (%)	No. of Isolates	Species (no.)
Beef	8	2 (25.0)	2	*E*. *coli*
Chicken	8	3 (37.5)	3	*E*. *coli*, *K*. *pneumoniae*, ECC
Mutton	8	1 (12.5)	1	ECC
Pork	8	2 (25.0)	2	*E*. *coli*
Clam	5	1 (20.0)	1	*E*. *coli*
Fish	8	1 (12.5)	1	ECC
Squid	8	0 (0)	0	−
Prawn	8	1 (12.5)	1	*K*. *pneumoniae*
Chinese cabbage	8	2 (25.0)	4	*E*. *coli*, *K*. *pneumoniae*, ECC, Sm
Lettuce	8	1 (12.5)	1	*E*. *coli*
Roselle	8	2 (25.0)	3	*E*. *coli*, *K*. *pneumoniae*, ECC
Water spinach	8	4 (50.0)	8	*E*. *coli* (4), *K*. *pneumoniae* (2), ECC (2)
Total	93	20 (21.5)	27

Resistant isolates possessed multiple genes encoding aminoglycoside modification enzymes, chromosomal point mutation in quinolone-resistance-determining regions of *gyrA* and *parC*, as well as plasmid-mediated quinolone-resistance determinants. Notably, we found the 16S rRNA methylase gene *rmtB* in nine isolates resistant to gentamycin and amikacin. In contrast, all isolates except one were susceptible to fosfomycin and colistin. The only isolate resistant to the latter antibiotic was classified as *Serratia*, known to be intrinsically colistin-resistant^11^. Multilocus sequence typing (MLST) classified 13 *E*. *coli* isolates into 10 sequence types (STs) and six *K*. *pneumoniae* isolates each into a different ST (Fig. 1). Further evidence of isolate diversity includes *bla*_NDM_ presence in differently sized plasmids or chromosomes (Fig. 1).

### Carbapenemase-producing *E*. *cloacae* complex possesses *bla*_IMI-1_

**Figure 2:**
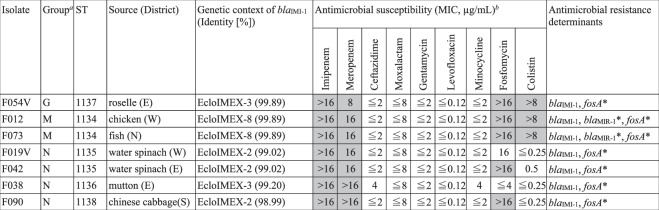
Phenotypic and genetic characteristics of *E*. *cloacae* complex isolates harbouring *bla*_IMI-1_. ^*a*^Phylogenetic groups were classified according to Chavda *et al*.^13^. ^*b*^Isolates resistant to the indicated antimicrobials are represented as grey shaded cells. MIC, minimum inhibitory concentration. *Genes with nucleotide substitutions compared with sequences in ResFinder.

Using the carbapenem-inactivation method (CIM), we identified seven carbapenemase-positive isolates of *Enterobacter cloacae* complex that are negative for *bla*_NDM_, *bla*_KPC_, *bla*_IMP_, or *bla*_OXA-48_ according to a PCR-dipstick test. Instead, whole genome sequencing revealed that they all possessed *bla*_IMI-1_, a chromosomally encoded Ambler class A serine β-lactamase gene. In accordance with an earlier report^12^, *bla*_IMI-1_ presence conferred high resistance to imipenem and meropenem, but not to extended-spectrum cephalosporin and oxacephem antibiotics, such as ceftazidime and moxalactam (Fig. 2). These bacteria were also susceptible to gentamycin, minocycline, and levofloxacin. Five isolates out of seven were resistant to fosfomycin, and three were resistant to colistin, a last-resort antibiotic for treating CPE infections.

**Figure 3:**
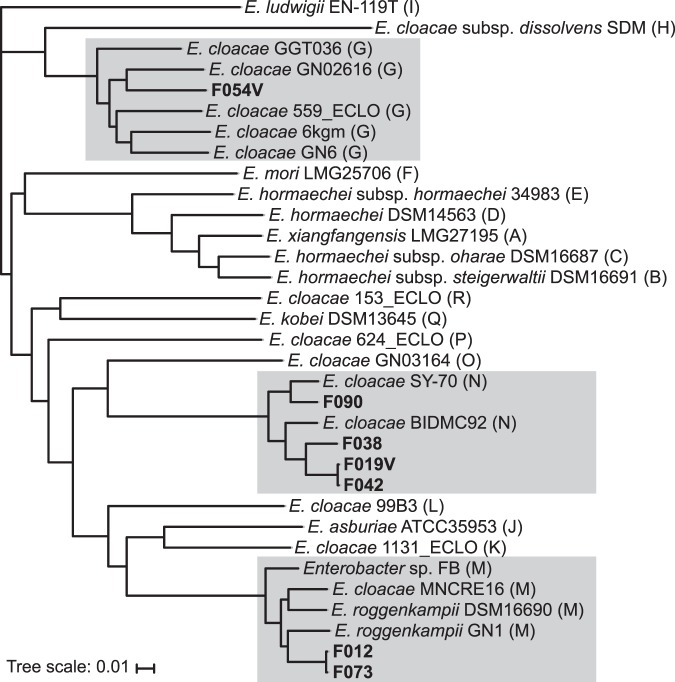
Phylogenetic analysis based on core-genome SNPs separates market-food-derived IMI-1-producing isolates into groups G, M, and N of the *E*. *cloacae* complex. A phylogenetic tree was constructed using the genome of *E*. *cloacae* ATCC13047 (GenBank accession number NC_014121.1) as a reference. Genomic sequences of other *E*. *cloacae* complex strains were retrieved from NCBI and included in the analysis. The food isolates acquired in this study are in bold; strains belonging to groups G, M, and N are marked in grey.

These six isolates were newly assigned to five STs representing previously undetected populations. We used whole-genome sequencing data of these isolates and those from the GenBank database to generate a core-genome single-nucleotide-polymorphism-based phylogenetic tree of the *E*. *cloacae* complex (Fig. 3). This tree grouped the newly assigned isolates into three clusters (G, M, and N)^13^. Each isolate carried *bla*_IMI-1_ between *setB* and *yeiP*; the gene was encoded on an integrative mobile element designated as EcloIMEX^14^. We identified three mobile elements, EcloIMEX-2 (GenBank accession number KR057494.1), -3 (KU870977.1), and -8 (MG547711.1). Of these, EcloIMEX-3 was found in phylogenetically different isolates, F038 and F054V.

## Discussion

This study found diverse CPE in local market foods with higher isolation rates than previously reported (2.4%)^8^. Vegetables were the most contaminated, exhibiting the highest CPE-positive rate (28.1%) and containing approximately 60% of isolates identified. Following vegetables, meat samples had high isolation rates (25.0%).

Carbapenem-resistant *E*. *coli* and *K*. *pneumoniae* isolates exhibited multidrug resistance because they harboured *bla*_NDM-1_ or *bla*_NDM-5_, along with other antimicrobial-resistance determinants. Worryingly, most isolates possessed *bla*_NDM-5_, which confers higher resistance against carbapenems than *bla*_NDM-1_^15^. Furthermore, we found evidence that *E*. *coli* isolates harbouring *bla*_NDM_ have clinical links; five of the 10 STs identified had been previously detected in a tertiary-care hospital of Yangon^10^. In contrast, NDM-producing *K*. *pneumoniae* isolates belonged to STs that have not been detected thus far from Yangon clinical specimens, although four (ST15, 16, 39, 395) have been found in clinical isolates from other countries^16,17^. Notably, we identified isolate F069 in chicken and showed that it was resistant to all tested antibiotics except colistin. This strain belongs to ST395, a constituent of worldwide epidemic clonal group 258^18^.

Isolation of IMI-type carbapenemase is relatively rare. Here, we provided valuable empirical evidence of diverse IMI-producing *E*. *cloacae* complexes existing outside hospitals, confirming previous long-term clinical observations that suggested that these bacteria are present in local communities^14,19^. Moreover, we confirmed the presence of IMI-type carbapenemase in Myanmar, suspected ever since *bla*_IMI-2_ was detected in a Dutch individual travelling to the country^20^.

We linked *bla*_IMI-1_ to a putative mobile element EcloIMEX in all isolates containing the gene. Among the three EcloIMEX detected, EcloIMEX-3 was found in genetically different group N (F038) and G (F054V) isolates, suggesting independent acquisition of the element. Thus, EcloIMEX is probably responsible for mobilizing *bla*_IMI_-type genes to *E*. *cloacae* complex^14,19^.

We isolated a *bla*_IMI-1_-harbouring *E*. *coloacae* complex from fish and water spinach, which is usually cultivated in water-rich regions, corroborating previous reports that associated these bacteria with aquatic environments^6,21,22^. Of note, we isolated two water spinach isolates of the same ST and EcloIMEX type from different districts, suggesting the existence of a shared reservoir of *bla*_IMI-1_ dissemination. Other sources are also likely, given the presence of *bla*_IMI-1_-harbouring isolates in other vegetables, as well as chicken and mutton, across all four surveyed districts.

Overall, our results demonstrate widespread dissemination of *bla*_IMI-1_-harbouring *E*. *coloacae* complex in Yangon. These organisms are susceptible to several common antibiotics, meaning that multiple treatment options are available in the event of infection such as reported previously^23,24^. However, they may sporadically become more resistant to drugs through acquiring multidrug-resistance plasmids in local communities. We therefore strongly recommend vigorous countermeasures to prevent pathogen spread through daily consumption of food stuffs in regions heavily contaminated by CPE and other multidrug-resistant microbes.

In conclusion, this study provided evidence that local market foods in Yangon, Myanmar frequently harboured diverse CPE isolates, some with highly drug-resistant phenotypes. We note two important limitations of our study: the small sample size and the possible existence of multiple sources for CPE spread. These factors prevented us from tracking exact CPE dissemination profiles in market food samples. Thus, further study is necessary to fully understand the extent of CPE dissemination in this region and to identify the major source(s) of their spread. Such knowledge will enable the development of effective countermeasures against contamination of food with CPE. Nevertheless, our research highlights the immediate need to take corrective action against the current situation of CPE contamination in food.

## Methods

### Isolation of carbapenemase-producing organisms from market food

Food sampling and bacterial isolation were conducted as previously described^25^. From November to December 2017, chicken, pork, beef, fish, clam, and assorted vegetables were collected from eight street stalls in Yangon. Samples were placed in an ice box and transported to the laboratory at the Department of Medical Research, Yangon, where they were either examined immediately or stored at 4 °C until further analysis. For sample processing, 25 g of flesh was mixed with 225 mL buffered peptone water in a stomacher bag and incubated at 35 °C for 18 ± 2 h. Subsequently, a loopful of enrichment culture was streaked onto CHROMagar ECC (CHROMagar, Paris, France), supplemented with 0.25 μg/mL meropenem and 70 μg/mL ZnSO_4_^26^, then cultured at 35 °C for 24 ± 2 h.

### Bacterial identification, antimicrobial-susceptibility tests, and PCR for carbapenemase genes

Bacteria were identified with matrix-assisted laser desorption/ionization time-of-flight mass spectrometry (MALDI Biotyper; Bruker Daltonics, Germany). Antimicrobial susceptibility was measured using a MicroScan WalkAway 96 Plus (Beckman Coulter, Brea, CA, USA) and dry plate (Eiken Chemical, Tokyo, Japan), then classified based on the Clinical and Laboratory Standards Institute Guidelines (M100-S24). For colistin, resistance was defined according to the European Committee on Antimicrobial Susceptibility Testing criteria. Carbapenem-resistant *Enterobacteriaceae* isolates were screened for carbapenemase production using CIM^27^. In addition, PCR-dipstick chromatography was performed to detect four major carbapenemase genes (*bla*_NDM_, *bla*_KPC_, *bla*_IMP_, and *bla*_OXA-48_)^28^.

### Whole-genome sequencing and plasmid analysis

Bacterial isolates were cultured overnight in brain-heart infusion broth (BD Bacto, Franklin Lakes, NJ, USA) containing 0.25 μg/mL meropenem. Genomic DNA was then prepared using a PowerSoil DNA isolation kit (Qiagen, Valencia, CA, USA). Library preparation was performed as described previously^9^ or using KAPA Hyper Plus Kits (KAPA Biosystems, Woburn, MA, USA). Paired-end sequencing with 250 bp reads was performed in MiSeq (Illumina, San Diego, CA, USA). Reads were then *de novo*-assembled using CLC Genomics Workbench 11.0.1 (CLC Bio, Aarhus, Denmark) for subsequent analyses.

Multilocus sequence typing for *E*. *coli* and *K*. *pneumoniae* were performed using EnteroBase (http://enterobase.warwick.ac.uk/) and the Institute Pasteur’s MLST database (http://bigsdb.pasteur.fr/klebsiella/klebsiella.html), respectively. Existing methods^29^ were followed for MLST of the *E*. *cloacae* complex. Resistance-gene identification was performed in ResFinder 2.1^30^. Evolutionary relatedness of isolates was assessed using CSI Phylogeny 1.4^31^, and iTOL was used to construct the phylogenetic tree^32^. To classify EcloIMEX, local BLAST searches were performed on sequences around *bla*_IMI-1_, which contain *setB* and *yeiP* genes.

To determine the *bla*_NDM_-harbouring plasmid size, pulsed-field electrophoresis (PFGE) plugs prepared from isolates were treated with S1 nuclease (Takara Bio, Shiga, Japan) and subjected to PFGE in the CHEF Mapper XA system (Bio-Rad, Hercules, CA, USA). Separated DNA was then transferred to a nylon membrane and probed with a digoxigenin-labelled (Roche Diagnostics, Basel, Switzerland), *bla*_NDM_-specific DNA probe for Southern hybridization.

## Data Availability

The sequence data were submitted to the DDBJ/GenBank/ENA database under BioProject number PRJDB5126.
